# Exercise Intervention in Treatment of Neuropsychological Diseases: A Review

**DOI:** 10.3389/fpsyg.2020.569206

**Published:** 2020-10-22

**Authors:** Zichao Chen, Wencen Lan, Guifen Yang, Yan Li, Xiang Ji, Lan Chen, Yan Zhou, Shanshan Li

**Affiliations:** ^1^Institute of Sport Science, Sichuan University, Chengdu, China; ^2^College of Applied Technology, Sichuan Normal University, Chengdu, China

**Keywords:** exercise intervention, neuropsychological disease, ADHD, depression, anxiety, autism

## Abstract

Faced with a constant inundation of information and increasing pressures brought by the continuous development of modern civilization, people are increasingly faced with mental health challenges that are only now being actively researched. Mental illness is caused by brain dysfunction due to internal and external pathogenic factors that destroy the integrity of the human brain and alter its function. Regular participation in physical exercise can stimulate the cerebral cortex and simultaneously increase the supply of oxygen and nutrients, helping to preserve or restore normal functioning of the nervous system. In conjunction with other systems of the body, the nervous system constitutes the neuro-humoral regulation system responsible for maintaining the stable state of the human body. This paper is a systematic review of studies investigating the effects of exercise intervention on several common neuropsychological diseases, including depression, anxiety disorder, autism, and attention-deficit/hyperactivity disorder. Furthermore, we discuss possible physiological mechanisms underlying exercise-induced benefits and study limitations that must be addressed by future research. In many cases, drug therapy is ineffective and brings unwanted side effects. Based on the literature, we conclude that exercise intervention plays a positive role and that certain standards must be established in the field to make physical activity consistently effective.

## Introduction

Neuropsychology investigates the relationships between brain processes and mechanisms on one hand and cognition and behavioral control on the other (Berlucchi, 2009). The rapid development of modern society has improved people’s living standards but has also taken a toll on their physical and mental health. The mortality rate of patients with severe neuropsychological disorders is two to three times higher than that of the general population (Saha et al., 2007; Walker et al., 2015). A meta-analysis determined that 32.6% of patients with severe mental disorders also suffer from metabolic syndrome (Vancampfort et al., 2015). Co-morbidities, mainly cardiovascular disease, are found in about 60% of people who die from severe neuropsychological diseases (Lawrence et al., 2013).

Exercise is associated with a range of health benefits: it can improve physical as well as mental health. People who exercise generally report improved quality of life, reduced psychological stress, and improved physical function (Fiuza-Luces et al., 2013; Schuch et al., 2016b, 2015; Vera-Garcia et al., 2015). In addition, exercise intervention has almost no negative side effects. Conversely, the negative effects of lack of exercise are manifold. Harmful effects on health and personal well-being include increased incidence of coronary heart disease, diabetes, certain cancers, obesity, and high blood pressure (Booth et al., 2012). Cross-sectional and prospective longitudinal studies have shown that a lack of physical activity is associated with depression and anxiety symptoms (Bhui and Fletcher, 2000; [Bibr B71][Bibr B1]; Haarasilta et al., 2004; Motl et al., 2004).

According to studies on both animals and humans, physical exercise can bring lasting benefits, such as improved cognitive function, increased cerebral blood flow, reduced oxidative stress response, increased neurotransmitter levels and plasticity, and improved ability to concentrate and process information (Radak et al., 2001; Ferris et al., 2007; Hillman et al., 2008; Stroth et al., 2009). Additionally, physical exercise can release stress (Tsatsoulis and Fountoulakis, 2006) and reduce negative psychology in patients with neuropsychological diseases, such as anxiety (Binder et al., 2004) and depression (McKercher et al., 2009; Lepage and Crowther, 2010).

Exercise training can improve the neurocognitive ability of patients with mental disorders, such as schizophrenia or depression (Oertel-Knochel et al., 2014; Greer et al., 2015). Given the great potential of exercise for improving physical and mental health, exercise could be developed specifically for cross-diagnosis and treatment of patients with neuropsychological disorders. Due to the current frequency and seriousness of mental diseases, an up-to-date and objective understanding of the therapeutic role of physical activity is needed.

Based on our findings, we put forth the recommendation that exercise may be exploited to reduce morbidity and mortality associated with mental illness. Our recommendation is based on some review of the available literature about the effects of exercise intervention on several of the most common neuropsychological diseases among children, adolescents, and adults in modern society (Mishra et al., 2018; Durbeej et al., 2019; Zablotsky et al., 2019): depression, anxiety disorder, autism, and attention-deficit/hyperactivity disorder (ADHD). We examined interventions, their possible therapeutic mechanisms, and limitations.

### Literature Search

**FIGURE 1:**
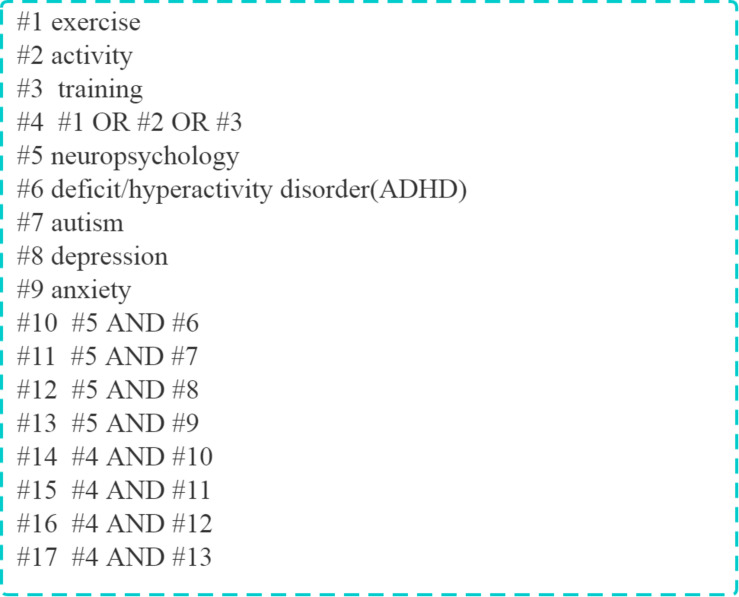
Search strings used to search the PubMed database.

The following electronic databases were systematically searched for relevant literature from inception to January 1, 2020: PubMed, Embase, the Cochrane Library, Web of Science, China Biology Medicine Disk, WanFang Data, and China National Knowledge Infrastructure. Eligible articles were also screened from reference lists of included studies. The following search strings were used: (“exercise” OR “activity” OR “training”) AND (“neuropsychology” OR “ADHD” OR “autism” OR “depression” OR “anxiety”). Additionally, the language restriction was set as English. An example of a retrieval strategy in PubMed is shown in Figure 1. A total of 167 publications were scrutinized.

## Common Neuropsychological Diseases and Exercise Interventions

### ADHD

Attention-deficit/hyperactivity disorder is one of the most common neurodevelopmental disorders in children and adolescents, with a prevalence rate of 8–10% worldwide (Colvin and Stern, 2015; Faraone et al., 2003; Polanczyk et al., 2007; Thomas et al., 2015). Symptoms develop over time and include inattention, excessive activity, and impulsiveness (Polanczyk et al., 2007). Over half (57%) of children diagnosed with ADHD struggle with symptoms into adulthood (Biederman et al., 2010; Fayyad et al., 2017), which severely impacts their individual learning, communication, normal life, and social ability (Coelho et al., 2010).

At present, ADHD is treated mainly with drugs (Ng, 2017) and behavioral or psychological intervention (Pliszka and Issues, 2007). Early administration of drugs or psychological intervention often adversely affects the child’s growth and development. For example, short-term use of stimulants may cause headaches, insomnia, anorexia, and nausea; long-term use may stunt growth (Greenhill et al., 2001; Hansen and Hansen, 2006; LeBlanc-Duchin and Taukulis, 2007; Scherer et al., 2010; Martinez-Raga et al., 2013; Childress and Sallee, 2014; Wang et al., 2013). Although stimulants such as methylphenidate can be effective, 20–25% of ADHD patients do not respond to such drugs (Childress and Sallee, 2014). Treatment duration and complexity can make life difficult for patients and their families (Jensen et al., 2007). ADHD treatment is marred by many deficiencies that must be urgently addressed through alternative therapies. Exercise offers a potential alternative as it is a natural and essential part of development.

#### Effects of Exercise Intervention on Children and Adolescents With ADHD

Cognitive ability and executive function appear to be hindered in ADHD patients, manifesting as lack of attention, forgetfulness, impulsiveness, and lack of organizational ability and perseverance (Willcutt et al., 2005; Diamond, 2013; Coghill et al., 2014; Silverstein et al., 2020). Indeed, evidence suggests that ADHD develops in patients as a result of a lack of executive function (Willcutt et al., 2005) and motivation (Nigg, 2005). The cognitive and executive ability of ADHD patients significantly improves with moderate- to high-intensity exercise, which may be indirectly reflected through improved academic performance (Castelli et al., 2007; Medina et al., 2010; Chang et al., 2012; Grassmann et al., 2017). In addition, physical exercise can reduce dependence on ADHD drugs (Katz et al., 2010). One review (Den Heijer et al., 2017) summarized several studies that revealed that physical exercise may represent an effective treatment option that could be combined with other treatment approaches for ADHD, but it highlighted that more well-controlled studies are needed in both children and adults. Therefore, exercise may directly and indirectly benefit ADHD-related mental and physical symptomology. Basic information regarding studies in recent years exploring the role of exercise intervention in patients with ADHD is shown in Table 1.

**TABLE 1:** Studies of the Effects of Exercise Intervention on Children with Attention-Deficit/Hyperactivity Disorder (ADHD).

Study	Sample(s)	Study Design	Age	Intervention	Period	Outcome Measurements	Result
Messler et al., 2018	28	RCTs	8–13 (male)	HIIT (3 times/wk, 25 min/session)	3 weeks	FBB-HKS, SBB-HKS	Concentration levels improved significantly.
Memarmoghaddam et al., 2016	40	RCTs	7–11 (male)	Walking, treadmill running, high jump, ball sports (3 times/wk, 90 min/session)	8–12 weeks	GHA, BSQ	Attention and behavior inhibition in the ADHD group were improved.
Benzing and Schmidt, 2017	66	RCTs	8–12	Shape up exercise (3 times/wk, 30 min/session)	13 weeks	The Conners-3 scales	Positive effects on the executive functions, sport motor performance. and ADHD symptoms.
Benzing and Schmidt, 2019	51	RCTs	8–12	Exergaming (3 times/wk, 30 min/session)	8 weeks	The Conners-3 scales	Exergaming benefited executive functions and motor abilities in children with ADHD.
Bustamante et al., 2016	35	RCTs	6–12	After-school exercise program (5 times/wk, 90 min/session)	10 weeks	STOPIT, AWMA-S	ADHD symptoms in children improved.

*AWMA-S, Automated Working Memory Assessment System Short Version; BSQ, Behavior Screening Questionnaire; FBB-HKS, German Version of the Questionnaire for External Evaluation by the Guardians; GHA, General Health Assessment; HIIT, high-intensity interval training; RCTs, randomized controlled trials; SBB-HKS, the form for self-assessment by the children; STOPIT, secondary executive function outcomes included the Stop Signal Inhibition Task.*

#### Possible Mechanism of Exercise Intervention in the Treatment of ADHD

Attention deficit/hyperactivity disorder may potentially benefit from exercise-related increases in noradrenaline (NE), dopamine (DA), and 5-hydroxytryptamine (5-HT) levels in the prefrontal cortex, hippocampus, and striatum (Smith et al., 2013; Verret et al., 2012). Norepinephrine is involved in the control of executive function and impulses (Robinson, 2012). DA is essential for normal motor and cognitive function of the brain (Cropley et al., 2006) and is found widely lacking in the prefrontal cortex of ADHD patients (Russell, 2002). Increases in 5-hydroxytryptamine and endogenous opioid peptide levels after exercise can further strengthen attention and emotional processing (Hillman et al., 2008). Therefore, exercise produces physiological effects similar to stimulant drugs used to treat ADHD, thereby alleviating symptoms.

### Depression

Depression is a life-threatening and disabling mental illness that is affecting more and more people around the world at an alarming rate (Global Burden of Disease Study, 2015). In extreme cases, depression can even compromise someone’s health more than physical disease (Moussavi et al., 2007). In patients suffering from somatic diseases, such as cancer, cardiovascular disease, and infection, depression further increases the risk of death. Not only can depression lead to emotional changes and reduced activity, but as many as two-thirds of patients also suffered from cognitive impairment—which can last even after symptoms have been alleviated (Behnken et al., 2010; Rock et al., 2014; Chakrabarty et al., 2016).

Cognitive defects are an important determinant of psychosocial function, and their persistence weakens the capacity for psychosocial rehabilitation (Reppermund et al., 2009). One meta-analysis concluded that cognitive impairment should be the core of diagnosis and treatment (Rock et al., 2014). Since psychotropic antidepressants have almost no regulatory effect on cognitive function in patients with depression (McIntyre et al., 2013), non-pharmacological methods are becoming increasingly important in the treatment of cognitive impairment.

#### Effects of Exercise Intervention on Depression

Compared to non-depressed patients, patients with depression initiate less physical activity, and their health deteriorates faster (Andersson et al., 2015). Exercise is positively correlated with improvement of mental health. A meta-analysis including 25 randomized controlled trials (RCTs) confirmed that exercise intervention is a good method for the treatment of depression (Schuch et al., 2016a), and it may also function as adjuvant therapy combined with antidepressant drugs (Kvam et al., 2016). Exercise intervention induces antidepressant effects among patients with depression (Cooney et al., 2013; Schuch et al., 2016a) that are, in some cases, comparable to those of antidepressant drugs or psychotherapy (Brosse et al., 2002; Blumenthal et al., 2007). Cognitive ability and other psychiatric indicators were improved in depressed patients after 4 weeks of aerobic endurance training (Oertel-Knochel et al., 2014), while a meta-analysis found that aerobic exercise significantly alleviated severe depressive symptoms in adults (Morres et al., 2019). In a study conducted on 13 adolescent patients with depression, gradual increases in aerobic exercise intensity over 12 weeks significantly alleviated depressive symptoms (Dopp et al., 2012). In this way, a large number of studies have confirmed that exercise intervention can reduce the symptoms of depression. Three meta-analyses about exercise intervention as treatment for depression are shown in Table 2. Considering the recurrent and serious effects of depression, timely understanding and full application of exercise intervention is necessary for better treatment.

**TABLE 2:** Three Meta-Analyses About Exercise Intervention as Treatment for Depression.

Study	Time Period Searched	Databases	Number of Studies	Sample (s)	Age	Design	Interventions	Outcome Measures	Meta-Analysis of Outcomes	Results
Schuch et al., 2016a	2013.01– 2015.08	ASP, MEDLINE, Psychology, BSC, PsycINFO, SPORTDiscus, CINAHL Plus, PubMed	25	1,487	18.4 to 76.4 (mean)	RCTs	Aerobic, resistance, mixed exercises	BDI, CSDD, GDS, HAMD, MADRS, MMPI, PHQ-9, SCL	SMD, 95% CI	Exercise has a large and significant antidepressant effect in people with depression.
Kvam et al., 2016	2007.01– 2014.11	SD, PsycINFO, MEDLINE, EMBASE, CENTRAL	23	977	18 to 69 (mean)	RCTs	Aerobic exercise, aerobic exercise + pharmacotherapy	BDI, CESD, HAMD, SCL-90	Hedges’s, 95% CI	Physical exercise is an effective intervention for depression.
Cooney et al., 2013	All years–2013.03	Cochrane Library, CENTRAL, MEDLINE, EMBASE, PsycINFO, SD	39	2,326	> 18	RCTs	Aerobic, resistance, aerobic+ resistance	BDI, HAMD	SMD, 95% CI	Exercise may be moderately more effective than no therapy for reducing symptoms of depression, but more evidence is needed.

*ASP, Academic Search Premier; BDI, Beck Depression Inventory; BSC, Behavioral Sciences Collection; CENTRAL, Cochrane Central Register of Controlled Trials; CESD, Center for Epidemiologic Studies Depression Scale; CSDD, Cornel Scale for Depression in Dementia; GDS, Geriatric Depression Scale; HAMD, Hamilton Rating Scale for Depression; MADRS, Montgomery–Asberg Depression Rating Scale; MMPI, Minnesota Multiphasic Personality Inventory; PHQ-9, Patient Health Questionnaire; SD, Sports Discus; SMD, standardized mean difference; SCL and SCL-90, Symptom Checklist; 95% CI, 95% confidence interval.*

#### Possible Mechanism of Exercise Intervention in the Treatment of Depression

Imaging studies show that structural changes in the hippocampus, amygdala, striatum, and frontal cortex—areas of the brain with high connectivity—are associated with early depression (Bjornebekk et al., 2005; Nguyen et al., 2019). The most consistent finding associated with depression is atrophy of the hippocampal region. Antidepressant drugs treat depression by promoting neurogenesis of the brain (Park, 2019). Likewise, exercise hypothetically promotes hippocampal neurogenesis through up-regulation of up to four factors: endorphins, vascular endothelial growth factor, brain-derived neurotrophic factor, and 5-hydroxytryptamine (Ernst et al., 2006).

Exercise may improve mood through other mechanisms as well. For example, exercise increases endocannabinoid levels, which are associated with analgesia, anxiety, and well-being (De Moor et al., 2006). Exercise is also associated with changes in the hypothalamic–pituitary–adrenal (HPA) axis, including an increase in corticotropin and a decrease in cortisol, two actions that together contribute to positive mood changes (Duclos and Tabarin, 2016). Finally, exercise improves the self-concept of patients with depression, and it may also promote the relief of depressive symptoms (Knapen et al., 2015).

### Anxiety Disorder

Anxiety disorder is a common, heterogeneous mental health disorder. Globally, the incidence of anxiety disorders in various countries ranges from 3.8% to 25%, and it is as high as 70% among people with chronic diseases (Remes et al., 2016). Anxiety disorders are divided into generalized anxiety disorder, social phobia, panic disorder, phobia, agoraphobia, separation anxiety disorder, and selective mutism (Kessler et al., 1994). These widespread mental diseases negatively impact people’s daily body function, quality of life, and health. In addition, anxiety disorders can easily coexist with other mental disorders, such as depression, which can hinder treatment (Kessler et al., 2008). Furthermore, anxiety disorders are associated with increased risk of cardiovascular disease (Tully et al., 2013; Batelaan et al., 2016) and premature mortality (Tully et al., 2008; Janszky et al., 2010).

Anxiety disorders are traditionally treated by pharmacotherapy (e.g., selective 5-hydroxytryptamine reuptake inhibitors and benzodiazepines) (Baldwin et al., 2005, 2011), cognitive behavioral therapy (Carpenter et al., 2018), or both (Ori et al., 2015). Although traditional therapy usually has good therapeutic effects, there are several downfalls. Nearly one-third of patients do not respond to treatment (Hofmann and Smits, 2008; de Vries et al., 2016), and even those who do may experience adverse side effects (Baldwin et al., 2005). Behavioral cognitive therapy is expensive and requires highly specialized professionals (Gunter and Whittal, 2010). Current treatments for anxiety disorders are not effective or even accessible to everyone; thus, more practical approaches should be explored.

#### Effects of Exercise Intervention on Anxiety Disorder

Exercise intervention is an effective treatment for a variety of mental diseases (Ahlskog et al., 2011; Firth et al., 2017; Gordon et al., 2017). For people with anxiety disorders, exercise intervention may be a promising, affordable, and accessible treatment option free of side effects. People who do not exercise reportedly suffer from a significantly higher risk of suffering anxiety and severity of panic disorder (Smits and Zvolensky, 2006). Sedentary lifestyles increase the risk of developing anxiety disorders (Teychenne et al., 2015) or other mental disorders such as depression (Schuch et al., 2018). Indeed, one study confirmed that the degree of anxiety in the exercise group was much lower than that in the control group that did not exercise (Wipfli et al., 2008). In addition, exercise training can reduce the anxiety symptoms of sedentary people with chronic diseases (Herring et al., 2010). Studies of the general population found that physically active people were at a lower risk of developing anxiety disorders or showed fewer symptoms of severe anxiety disorders (De Mello et al., 2013; Lindwall et al., 2014). Stonerock’s review and Bartley’s meta-analysis indicated that exercise may be a useful treatment for anxiety (Bartley et al., 2013; Stonerock et al., 2015). However, those analyses highlighted that definitive conclusions about the efficacy of exercise require additional rigorous, methodologically sound RCTs, larger samples, and comparisons that control for exercise time. Studies in recent years about exercise intervention in patients with anxiety disorders are summarized in Table 3.

**TABLE 3:** Studies of Exercise Intervention to Treat Anxiety Disorders.

Study	Sample(s)	Study Design	Age	Interventions	Duration	Outcome Measures	Results
Gaudlitz et al., 2015	47	RCTs	> 18	Treadmill running (3 times/wk, 30 min/session)	8 weeks	Ham-A	Both high- and low-intensity exercise can relieve anxiety symptoms.
Kwok et al., 2019	187	RCTs	> 18	Mindfulness yoga (90 min/session) SRTE (60 min/session)	8 weeks	HADS	Mindfulness yoga can be as effective as SRTE in improving anxiety symptoms.
LeBouthillier and Asmundson, 2017	48	RCTs	> 18	Aerobic exercise and resistance training (3 times/wk, 50 min/session)	4 weeks	SCID-5-RV	Aerobic exercise and resistance training can improve general psychological distress and anxiety.

*HADS, Hospital Anxiety and Depression Scale; Ham-A, Hamilton Anxiety Scale; STAI, State Trait Anxiety Inventory; SRTE, resistance training exercise; SCID-5-RV, Structured Clinical Interview for DSM-5, Research Version.*

#### Possible Mechanism of Exercise Intervention in the Treatment of Anxiety Disorder

Exercise intervention may regulate the stress response through the HPA axis or glucocorticoid circulation (Anderson and Shivakumar, 2013), increasing cell proliferation and levels of brain-derived neurotrophic factor responsible for reducing anxiety. Exercise intervention may also work by up-regulating the endogenous cannabinoid system. Circulating cannabinoids produce anti-anxiety effects by regulating other neurotransmitters such as DA (Tantimonaco et al., 2014). These data strongly argue that exercise intervention can play an important role in the treatment of anxiety disorders.

### Autism

Autism is a highly heritable disease that usually occurs in infancy and childhood and follows a stable process without remission. According to the new edition of the *Diagnostic and Statistical Manual of Mental Disorders* from the American Psychiatric Association, people with autism have deficiencies in social interaction and communication skills and show repetitive, restrictive, and stereotyped behavior patterns and interest in activities. All these characteristic manifestations have an important impact on a child’s growth and the daily life of their family. The number of people diagnosed with autism is rapidly growing around the world. In Chinese children under the age of 15, about 1.61% of children are affected along the autism spectrum (Wong and Hui, 2008). Compared with the general population, children and adults with autism have a higher risk of other mental and medical diseases (Bradley and Bolton, 2006; Bauman, 2010; Croen et al., 2015), such as obesity and cardiovascular disease (McCoy et al., 2016). Autistic patients also have lower health indices than their normally developing peers, including cardiovascular endurance, upper and abdominal muscle strength, endurance, and lower limb flexibility (Pan et al., 2016). Surveys from parents reveal that their autistic children engage in significantly fewer types of physical activities and for less time annually than do their normal peers (Bandini et al., 2013).

The main purpose behind the treatment of people with autism is to improve their quality of life and reduce related defects and family suffering. Treatment is usually based on the needs of the child, but there is no single treatment that is sufficient to control all symptoms (Myers et al., 2007). Currently, there are no known medications that can relieve the core symptoms of autism, especially communication and social disorders (Myers et al., 2007). Although there is still no cure for autism, some treatments and interventions can help autistic children with daily functioning.

#### Effects of Exercise Intervention on Autism

Physical activity is especially important for children because it can not only strengthen their bodies but also improve their self-esteem, social skills, and behavior (Fisher et al., 2011). Conversely, many studies have pointed out the potential problems that can arise due to lack of exercise, especially for children with disabilities (Dobbins et al., 2013; Donnelly et al., 2016; Saunders et al., 2016; Pate et al., 2019). Due to the lack of social and communication skills, autistic children have little opportunity to play with their peers and participate in physical activities (Pan and Frey, 2006). Participation in physical activity allows autistic children to experience interesting activities with their peers and develop interpersonal skills (Srinivasan et al., 2014). Moreover, specific patterns of exercise intervention positively impact the social and communication skills of autistic children and increase their rapid response and frequency of expression (Zhao and Chen, 2018). In one study, ball games, fun games, and orienteering games all improved the perceptual motor skills of autistic adolescents (Rafie et al., 2017). In another study, 12 weeks of ping-pong training significantly improved the motor skill proficiency and executive function of autistic children (Pan et al., 2017). Although high-intensity aerobic exercise may aggravate the stereotypical behavior of autistic children, low- to moderate-intensity exercise can significantly reduce the occurrence of stereotyped behavior (Schmitz Olin et al., 2017). Basic information from recent studies about exercise intervention in patients with autism is shown in Table 4. Overall, exercise intervention appears to benefit the development of physical health and social communication skills, including operating skills, motor skills, muscle strength, and endurance for youth with autism (Healy et al., 2018). These studies establish that exercise intervention may be a feasible treatment for autism.

**TABLE 4:** Studies on Exercise Intervention in the Treatment of Autism.

Study	Sample(s)	Study Design	Age	Interventions	Duration	Outcome Measures	Results
Phung and Goldberg, 2019	34	RCTs	8–11	Mixed martial arts training (2 times/wk, 45 min/session)	13 weeks	SCQ, ADOS-2, WASI-II	The intervention appeared to be efficacious in meeting its goals of improving the executive functioning of children with ASD.
Sarabzadeh et al., 2019	18	RCTs	6–12	Tai chi chuan training (3 times/wk, 60 min/session)	6 weeks	GARS2	Tai chi chuan can improve balance and motion coordination.
Tse et al., 2019	50	RCTs	9.95 (mean)	Basketball skill learning (2 times/wk, 45 min/session)	12 weeks	CBTT, FDS test, BDS tests	Cognition among children with ASD was improved.
Sotoodeh et al., 2017	29	RCTs	7–15	Yoga (3 times/wk, 30 min/session)	8 weeks	ATEC	Yoga training can decrease the severity of autism.
Toscano et al., 2018	64	RCTs	6–12	Basic coordination and strength exercises (2 times/wk, 40 min/session)	48 weeks	CHQ-PF50, CARS	Basic coordination and strength exercises are important therapeutic interventions for children with ASD.

*ADOS-2, Autism Diagnostic Observation Schedule—Second Edition; ASD, autism spectrum disorder; ATEC, Autism Treatment Evaluation Checklist; BDS, backward digit span; CARS, Childhood Autism Rating Scale; CBTT, Corsi block tapping task; CHQ-PF50, Child Health Questionnaire; FDS, forward digit span; GARS2, Gilliam Autism Rating Scale—second edition; SCQ, Lifetime Social Communication Questionnaire; WASI-II, Wechsler Abbreviated Scale of Intelligence—Second Edition.*

#### Possible Mechanism of Exercise Intervention in the Treatment of Autism

Autism involves structural defects in the brain, including a decrease in forebrain volume and a disruption of neural networks between the limbic system and other cortical regions (Sairanen et al., 2005). Exercise intervention can increase the volume of hippocampal tissue and promote the production of nerves and blood vessels in patients with autism by increasing brain-derived neurotrophic factor in the cerebral cortex (Vaynman et al., 2004). It can also promote the production of neurotrophic factors, including nerve growth factor and fibroblast growth factor-2, which can improve the neuropsychological function of autistic children (Courchesne et al., 2001; Croen et al., 2008). In animal models of autism, exercise intervention stimulated the signaling pathway involving phosphatidylinositol-3-kinase (PI3K), protein kinase B (Akt), and extracellular signal-regulated protein kinases 1 and 2 (ERK 1/2), leading to inhibition of neuronal apoptosis in the brain and thereby improving spatial learning, memory, and decision making as well as neurogenesis in the hippocampus (Seo et al., 2013). In addition, exercise intervention improves the cognitive ability of people with autism and reduces repetitive behaviors (Anderson-Hanley et al., 2011). Other evidence shows that exercise intervention can enhance memory function in people with autism (Chan et al., 2015). In summary, studies support exercise intervention for mitigating the symptoms of autism.

## Potential Mechanisms of Exercise Intervention in Improving Neuropsychological Diseases

**FIGURE 2:**
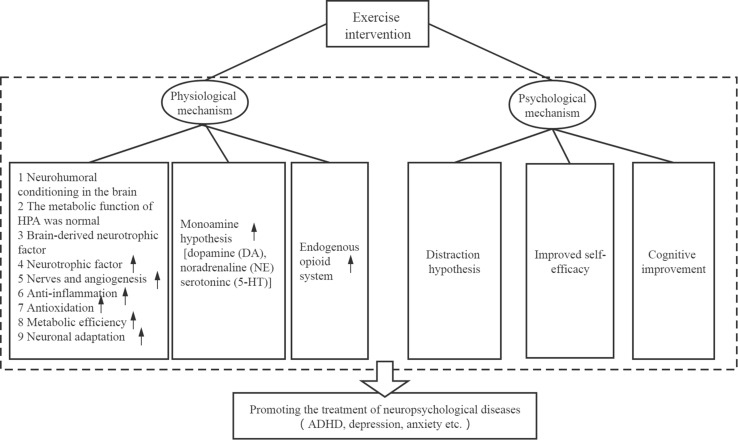
Possible therapeutic mechanisms.

Exercise intervention is hypothesized to alleviate symptoms of neuropsychological diseases through a series of different physiological and psychological mechanisms. These mechanisms are shown in Figure 2 and described in greater detail below.

### Proposed Physiological Mechanisms

Neuro-humoral regulation in the brain is one proposed physiological mechanism. Regular aerobic exercise is associated with lower activity of the sympathetic nervous system and HPA cascade (Jackson and Dishman, 2006; Rimmele et al., 2007). The HPA cascade plays a key role in coping with physical and psychological stress, whereas a disordered reaction is related to the occurrence of depression and anxiety (Swaab et al., 2005). Exercise changes the release of corticotropin-releasing factor in the hypothalamus and corticotropin in the anterior pituitary (Salmon, 2001; Droste et al., 2003). These findings suggest that changes in the HPA cascade response induced by exercise can regulate the stress response, anxiety, and depression in humans.

Exercise intervention can also stimulate a series of neurogenic processes that are important for normal function of the brain: it up-regulates growth factors and brain-derived neurotrophic factors, stimulating neurogenesis and angiogenesis (Voss et al., 2013; Kandola et al., 2016). Brain-derived neurotrophic factor is the most abundant neurotrophic factor in the brain, and it is related to anxiety and depression. Stress-induced depression and anxiety behavior are associated with decreased levels of neurotrophic factors in the brain, especially in the hippocampus (Duman and Monteggia, 2006). People with depression have reduced hippocampal volume (Campbell et al., 2004), and 3 months of regular exercise can increase hippocampal volume and improve memory (Pajonk et al., 2010). It is indeed possible that physical activity has a direct positive effect on the hippocampus.

The monoamine hypothesis holds that exercise enhances brain aminergic synaptic transmission (Swaab et al., 2005). DA, NE, and serotonin (5-HT) are the three major monoamine neurotransmitters that are known to be modulated by exercise (Lin and Kuo, 2013). Antidepressant medications are thought to work by improving aminergic transmission, which appears to be impaired in depressive disorders due to defects in production, transmission, reuptake, or metabolism (Pajonk et al., 2010). Another hypothesis postulates that exercise activates the endogenous opioid system. Endogenous opioids can regulate emotion and emotional response (Bodnar and Klein, 2006). Exercise-induced increases in activity of endogenous opioids in the central and peripheral nervous system simultaneously cause feelings of euphoria and relieve pain (Dinas et al., 2011).

Some studies revealed that antioxidant indicators tended to increase and pro-oxidant indicators tended to decrease after exercise training (de Sousa et al., 2017). Exercise training also has been shown to enhance brain function and ameliorate brain disorders by inducing neuroplasticity, increasing metabolic efficiency, enhancing neuronal adaptation, and improving anti-oxidative capacity (Gomez-Cabrera et al., 2008; Lin et al., 2012; Nagamatsu et al., 2014). Moreover, physical exercise can modulate microglial activation in the central nervous system and thereby prevent neuroinflammation in the central nervous system (Mee-Inta et al., 2019). Thus, it is suggested that people, regardless of their health condition, participate in certain kinds of exercise in order to balance the redox state and improve health-related outcomes.

### Proposed Psychological Mechanisms

Exercise can make us forget the troubles of daily life for a period of time, which supports the distraction hypothesis. In this theory, diverting attention from unpleasant stimuli or painful physical complaints via exercise can improve mood or simulate an antidepressant-like effect (Carek et al., 2011; Crush et al., 2018). In contrast, the popular “self-efficacy theory” originally proposed by Bandura (Bandura, 1977) suggests that a person’s sense of self-efficacy is positively correlated with his/her ability to control potential threats. Those who believe in their ability to manage potential threats (high self-efficacy) will not be bothered by worrying thoughts or experience low-level anxiety. In essence, if a treatment can rebuild the sense of self-efficacy by providing the experience of self-control, then it will succeed. Exercise itself can improve self-efficacy by providing experience in successfully dealing with stress (Guszkowska, 2004). In one study, the score of self-efficacy was closely related to the current exercise stage, while people who did not exercise lacked confidence in their exercise ability (Jonsson et al., 2018). Improvement in physical fitness results in more endurance and less pain, and it may contribute to positive ideologies. Successful regular physical activity can improve mood, enhance self-confidence, and enhance the ability to deal with challenging mental health events (Chu et al., 2014). These studies have shown that appropriate exercise intervention can improve self-efficacy.

## Limitations of Exercise Intervention

### Formulating Appropriate Types and Intensities of Exercise Interventions Against Different Psychological Disorders

Studies of exercise intervention may give different results depending on the type of training (e.g., running, walking, or cycling), as well as its duration and intensity. Future research should maintain a consistent and strict definition of aerobic exercise, which would greatly benefit the implementation of correct and effective exercise intervention. A good starting point might be the guidelines proposed by the American Heart Association: lower limb endurance training for 20–60 min, three to five times a week (Fletcher et al., 2001). Then intervention programs could be specifically tailored to suit the needs of individuals with different neuropsychological diseases.

However, an important caveat to consider is that excessive physical activity may be harmful to physical and mental health. Overtraining may cause a neurophysiological disorder and is associated with hyperglucocorticoidemia and hypothalamic dysfunction caused by insulin-induced hypoglycemia (Raglin, 1990; Angeli et al., 2004). Furthermore, excessive exercise decreases libido, retards psychomotor function, and induces other depressive states (Morgan et al., 1987). The boundary between adequate and excessive exercise patterns can be subtle for many people, so future research should examine how to define training intensities for different populations. In the case of ADHD patients, many studies have examined the effectiveness of only short-term exercise intervention in improving behavioral symptoms and neuropsychological function. Future research should include long-term aerobic training and measurements of executive function in order to confirm the long-term effectiveness of exercise intervention.

### Considering Patient Differences and Objective Measures

Most experimental designs have not taken into account the influence of gender and age, both of which may be associated with the risk of mental disorders. For example, the incidence of ADHD in boys (7.9%) is four times higher than that in girls (1.8%) (Schlack et al., 2007).

Considering the pessimism of patients with depression and the fact that exercise demands time and energy, many patients seem reluctant to participate in studies of how exercise may benefit them. In fact, lack of interest is a key symptom of depression and the main obstacle to treatment of mental illness more generally (Legrand and Neff, 2016). Clinical staff may find it difficult to stimulate a patient’s interest in exercise. This means that the results obtained in exercise studies of volunteers with depression cannot be broadly extrapolated to all people with depression.

Most studies of exercise intervention against psychological disorders have not applied an acceptable standardized set of measures, they often lack control groups, and they suffer from methodological bias. For example, clinical scoring relies heavily on self-report by the subjects, making it impossible to rule out subjectivity. The best way to minimize this potential deviation is to apply double blinding, but few studies have applied this design.

Future studies should examine whether particular subgroups of patients are more likely than others to benefit from exercise interventions. It may be necessary to optimize interventions for a given disease based on patients’ clinicodemographic characteristics.

## Conclusion

The universality of neuropsychological diseases highlights the necessity of diversified treatments. Pharmacological or behavioral interventions are not appropriate or effective for many patients. Clinical and animal studies and meta-analyses strongly support the benefits of exercise intervention for alleviating neuropsychological symptoms and overall disease. These positive impacts occur via several physiological and psychological mechanisms. Especially since people with neuropsychological disorders are at significantly greater risk of potentially serious co-morbidities (Tyler et al., 2011), such as obesity in autistic children, more work is urgently needed to study and establish exercise intervention as a standard of care in the treatment of neuropsychological diseases and coexisting health problems.

## Author Contributions

WL conceived the study. WL and ZC searched the literature and selected studies to analyze closely. GY and WL drafted the manuscript, which all authors revised and approved for publication.

## Conflict of Interest

The authors declare that the research was conducted in the absence of any commercial or financial relationships that could be construed as a potential conflict of interest.
